# An extraction from Trametes robiniophila Murr. (*Huaier*) inhibits non-small cell lung cancer proliferation via targeting to epidermal growth factor receptor

**DOI:** 10.1080/21655979.2022.2066757

**Published:** 2022-04-26

**Authors:** Fei Lv, Xiaoqi Li, Ying Wang

**Affiliations:** a Department of Oncology, Shengjing Hospital of China Medical University, Shenyang, Liaoning, China; b Third Department of Oncology, The People’s Hospital of Liaoning Province

**Keywords:** Molecular docking simulation, carcinoma, non-small-cell lung, traditional Chinese medicine

## Abstract

An extraction from Trametes robiniophila Murr. *(Huaier)* is a kind of natural fungus growing from the sophora japonica tree. *Huaier* is widely applied to cure the hepatocellular cancer (HCC). However, the medicinal fungus’ curative result on non-small-cell lung cancer (NSCLC) is not well elaborated. In this study, we applied *in vitro* experiments to study *Huaier’*s curative result on NSCLC. The potential *Huaier* targets were predicted using bioinformatics and validated by western blotting. We further elucidated the mechanism of *Huaier* targeting by molecular docking, kinase activity assay, CEllular Thermal Shift Assays (CETSAs). At last, *in vivo* curative result was verified further. *Huaier* weakened proliferation and promoted apoptosis of the NSCLC cell lines. According to bioinformatics, Epidermal Growth Factor Receptor (EGFR) may be the target of *Huaier*. Western blotting showed that *Huaier* can attenuate the activation of EGFR and we found that *Huaier* can dock to EGFR. *Huaier* significantly inhibited the tumor growth by weakening the expression of p-EGFR *in vivo*. This study offers a new idea for further understanding of *Huaier* and shows its potential as a therapeutic agent.

## Graphical abstract

**Figure:**
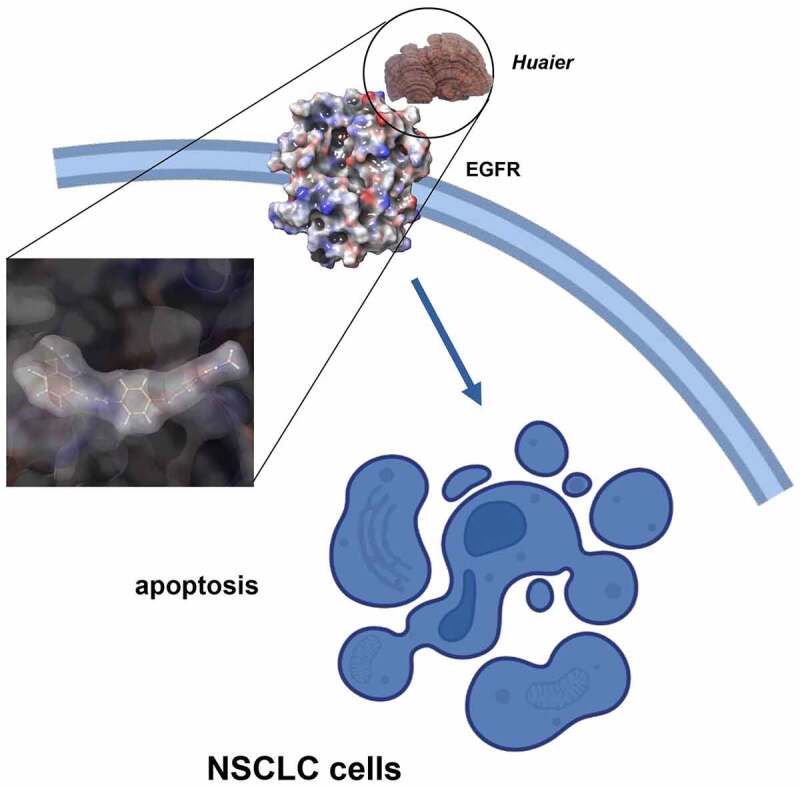

## Introduction

Eighty percent of lung cancer-related deaths are due to non-small-cell lung cancer (NSCLC) [1]. Chemotherapy is the preferred therapeutic method for advanced NSCLC, although its median overall survival (OS) does not exceed the upper limit of approximately one year [2]. The Epidermal Growth Factor Receptor – Tyrosine Kinase Inhibitor (EGFR-TKI), such as gefitinib, significantly extend survival and improve quality of life in NSCLC patients [3]. However, drug resistance limits the use of these drugs [4]. Therefore, the search for more effective and safe therapeutic drugs has become a hot topic in NSCLC research.

Traditional Chinese medicine cancer treatments have the advantages of few adverse effects, high safety, and improved quality of life [5,6]. *Huaier* is an extraction from Trametes robiniophila Murr., the main ingredient of which is a polysaccharide protein (PS-T), containing about 40% of polysaccharides, 10% of amino acids, six types of monosaccharides, and 18 amino acids [7]. In a prospective study of more than 1,000 patients, Chen [8] confirmed the curative result of *Huaier* granules as adjuvant therapeutic method for hepatocellular cancer (HCC) after hepatectomy. Patients who received *Huaier* granule adjuvant therapy were more prone to relapse-free survival (RFS) and overall survival (OS) than those who received placebo. Another study showed that liver cancer patients treated with *Huaier* experienced significantly prolonged duration recurrence time compared with those with transcatheter arterial chemoembolization (TACE) treatment alone [9]. Using microarray analysis, Wang [10] found that lncRNA H19 expression was down-regulated after breast cancer cell lines were administered with *Huaier*. Other studies have shown MAPK [11], Twist [12], and Wnt [13] are also *Huaier* targets. Besides, it also showed that *Huaier* inhibits triple-negative breast cancer metastasis [14]. However, the precise mechanism remains unknown.

Bioinformatics is a research method used in the identification of new drug targets based on a network perspective [15]. In pharmacological research of traditional Chinese medicine (TCM), bioinformatics can integrate the complex components, targets, and diseases. Here, PharmMapper and other databases were used to study the *Huaier* targets for the first time, revealing EGFR as the main target. A good spatial and energy match was found between *Huaier* and EGFR using molecular docking.

In this study, we explored the anti-cancer roles of *Huaier in vitro* and *in vivo* and the *Huaier* targets were predicted and verified. *Huaier* inhibited EGFR activity by binding to them directly. This study sharpened the understanding of *Huaier*’s molecular biological function and indicated that *Huaier* may be a promising adjunct therapy for NSCLC.

## Methods

### Cell lines and reagents

Human NSCLC cell lines A549 and H1299 were purchased from Cell Bank of Representative Culture Preservation Committee of Chinese Academy of Sciences (Shanghai, China). The NSCLC cell lines were cultured in standard cell culture medium and kept in an incubator at 37°C with 5% CO_2_.

*Huaier* stock solution was prepared as described before [16]. Adenosine was purchased form Sigma-Aldrich (A9251). Gefitinib and Daucosterol were both purchased from MCE (HY-50895; HY-N0410).

### MTT

Cell viability was examined using the 3-(4,5-dimethylthiazol-2-yl)-2,5-diphenyltetrazolium bromide (MTT) assay as described before [17]. Briefly, cells were seeded, exposed to *Huaier* (and/or Gefitinib), added MTT solution and measured using a spectrophotometer (BIOBASE, EL10A).

### Colony formation

Colony formation was conducted as described before [17]. The formed colony under *Huaier* (and/or Gefitinib) administration was stained and photographed with a digital camera and analyzed using ImageJ (National Institutes of Health (NIH), Bethesda, USA).

### Flow cytometry

We measured changes in apoptosis and cell cycle after *Huaier* (and Gefitinib) administration by flow cytometry and the data were analyzed using FlowJo (Version X; TreeStar, Ashland, OR) and ModFit LT (Verity Software House; Topsham, ME).

### EdU staining

The effect of *Huaier* on DNA replication was measured through EdU/Apollo488 staining (Ribobio, C10301-1), which was conducted as manufacturer’s protocal. Images were acquired by fluorescence microscope and analyzed by ImageJ.

### Bioinformatics prediction

Five *Huaier* constituents were previously reported [18]: adenosine, ergosta-7.22-dien-3 beta-ol, ergosteral, 3beta-hydroxystigmast-5, 22-dien-7-one, and daucosterol. Structural formulas for the five active components of *Huaier* were drawn using Chemdraw and uploaded to PharmMapper [19] (http://59.78.96.61/pharmmapper/) to obtain possible *Huaier* targets. The target names were then corrected to the official symbol using UniProt (https://www.uniprot.org/). Gene ontology (GO) functional annotation [20] and Kyoto Encyclopedia of Genes and Genomes (KEGG) pathway analysis [21] of the *Huaier*-related targets were performed using WebGestalt (http://www.webgestalt.org/) [22]. Lung cancer-related targets were obtained using CooLGen (http://ci.smu.edu.cn/CooLGeN/) and compared with the predicted targets to obtain possible *Huaier* targets for inhibition of lung cancer. Finally, the target names were submitted to the Cytoscape software [23] to visualize the protein–protein interaction (PPI) networks.

### Western blotting

Western blotting was done according to the protocal described before [17]. Proteins treated with different concentrations of *Huaier* were extracted, separated by electrophoresis, transferred to membranes, blocked and incubated with antibodies. Finally, membranes were visualized using the chemiluminescence reagent and densitimetric analysis was done by ImageJ.

### Molecular docking

Molecular docking was conducted using Schrödinger software. EGFR crystal structure (PDB ID: 2ITY) [24] were prepared using RCSB database [25] and optimized with the Protein Preparation Wizard module. Different conformational states for *Huaier* ligand molecules was generated by Ligprep module. Glide module [26,27] was applied to calculate the docking between *Huaier* and EGFR in standard precision (SP).

### EGFR enzymatic assay

We performed EGFR enzymatic assay using the Kinase Activity Assay Kit (Abace, Beijing, China), following the manufacturer’s protocol. We treated EGFR protein with different concentrations of *Huaier* (or adenosine, daucosterol) and measure the fluorescence intensity by an automatic microplate reader.

### CElluar thermal shift assays

We conducted CETSAs to reveal the binding between *Huaier* and EGFR *in celluar*. Briefly, cells, exposed to *Huaier* beforehand, were transferred into 1.5 ml PCR tubes and heated. Next, cells were lysed, separated then detected by western blot assays.

### In vivo *experiments*

4–6 weeks old BALB/c nude mice were purchased from the National Laboratory Animal Center (Shanghai, China). After 6 days of acclimatization, we injected 5*10^6^ A549 cells in the flank of each nude mouse subcutaneously. We assigned randomly the mice to vehicle control (saline) or *Huaier* alone (five animals in each group, 50 mg *Huaier*/100 µL solution) groups when the tumor volume reached approximately 60 mm^3^. We administered the drug gavagely once every three days and calculated tumor volume from the width and length of tumor body and sacrificed the mice after 21 days to remove their xenografts for immunohistochemical staining. The liver and kidneys in each group were dissected and embedded in paraffin to prepare sections for the hematoxylin and eosin (H&E) staining. In another experiment panel, mouse survival was evaluated. The study was performed in accordance with the Guidelines for Care and Use of Laboratory Animals of ‘National Institutes of Health’ and approved by the Ethics Committee of Shengjing Hospital of China Medical University (Supplementary Material 1).

### Immunohistochemistry

Immunohistochemistry was performed according to standard protocal. Briefly, tissue sections were prepared to remove the endogenous peroxidase, block with goat serum, incubate antibodies before colorating by 3,3’-diaminobenzidine tetrahydrochloride (DAB) kit (Maixin, China, DAB-0031). The expression of p-EGFR (Cell Signaling Technology, 8543S) and Ki-67 (Cell Signaling Technology, 9499) were assessed.

### Statistical methods

We repeated all experiments independently at least three times and analyzed results by SPSS software (SPSS, Chicago, IL, USA). The data were presented as the mean ± Standard Deviatio (SD) and standardized. We determined statistical significance between *Huaier* and control groups using student’s two-tailed t-test and one-way ANOVA. *In vivo* experiments, survival was evaluated using the Kaplan–Meier method. Differences were considered significant when p-values < 0.05.

## Results

In this study, we examined *Huaier*’s anti-NSCLC effects *in vitro* and *in vivo*, then explored it’s specific mechanism by molecular docking, kinase activity assay and CETSAs. We found that *Huaier* killed NSCLC cells by binding to EGFR directly. These findings provide new insights into the treatment of NSCLC.

### Huaier *inhibits NSCLC cell proliferation and promotes apoptosis*

**Figure 1.**
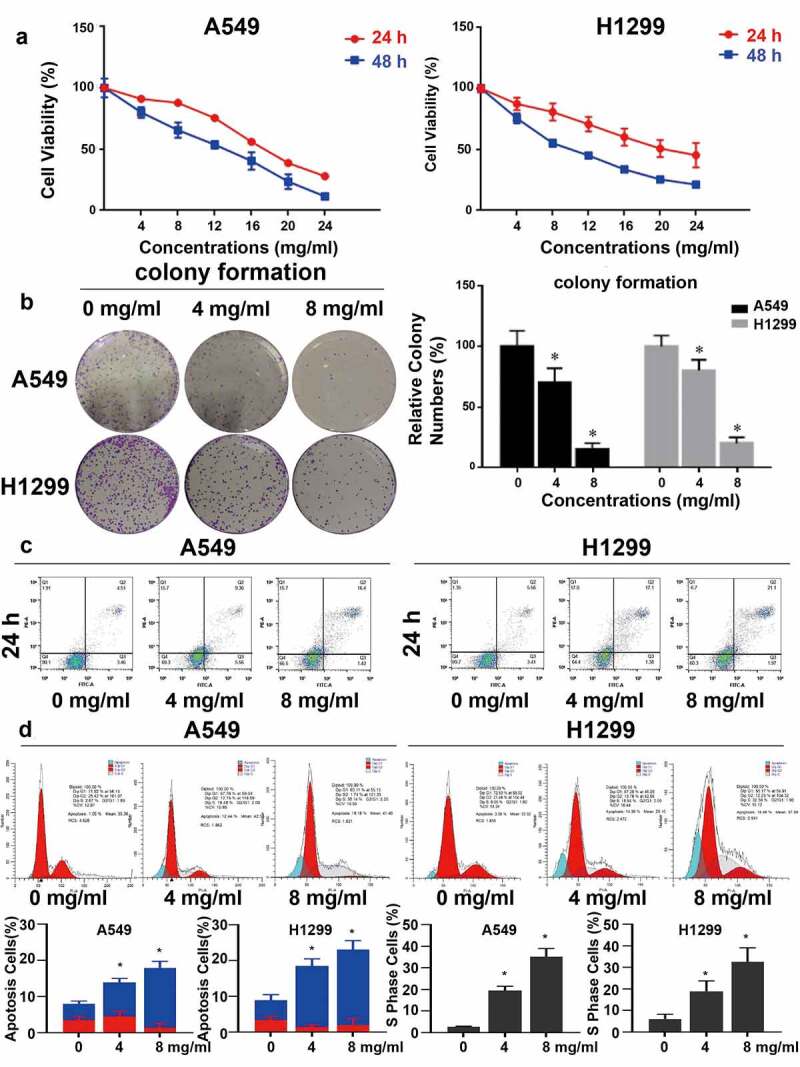
*Huaier* inhibits NSCLC growth *in vitro*. (a) *Huaier*’s inhibitory effect on NSCLC cell lines. A549 and H1299 cell lines were treated with *Huaier* and MTT assay was used to analyze the cell viability. (b) *Huaier* inhibited colony formation significantly on NSCLC cell lines. We treated the cell lines with *Huaier* before analyzing the colony formation. Data represents mean ± SD, *p < 0.05, n = 3. C, Flow cytometry analysis. We analyzed the apoptotic intensity by flow cytometry after the cell lines were treated with *Huaier*. D, *Huaier* arrests cell cycle. *Huaier*’s effect on NSCLC cell lines’ S phase population. Cell cycles were analyzed by flow cytometry and quantitatively analyzed after treated with *Huaier*. Histograms of flow cytometry results are presented at the bottom. Data represents mean ± SD, *p < 0.05, n = 3. The raw data of flow cytometry contained in Supplementary Material 3.

Different concentrations of *Huaier* were added to lung cancer cell lines to test inhibitory activity of *Huaier* on proliferation. MTT assay was applied to determine the cell viability of *Huaier* at different time points and at different concentrations, to detect *Huaier*’s inhibitory effect on the proliferation of lung cancer cells. As displayed in Figure 1(a), *Huaier* inhibited proliferation of lung cancer cells concentration- and time-dependently. We further conducted colony formation to determine *Huaier*’s anti-proliferation effect. As displayed in Figure 1(b), *Huaier* effectively inhibited colony formation. Then, we performed flow cytometry to test whether *Huaier* caused changes in the apoptotic cell number. Addition of the *Huaier* increased apoptotic cells and promoted late apoptosis (late apoptosis rate: A549: 0 mg/ml: 4.51% ± 0.8%, 4 mg/ml: 9.36% ± 1.1%, 8 mg/ml: 16.4% ± 1.9%. H1299: 0 mg/ml: 4.21% ± 0.9%, 4 mg/ml: 6.64% ± 1.4%, 8 mg/ml: 9.22% ± 2.0%. Figure 1(c)).

**Figure 2.**
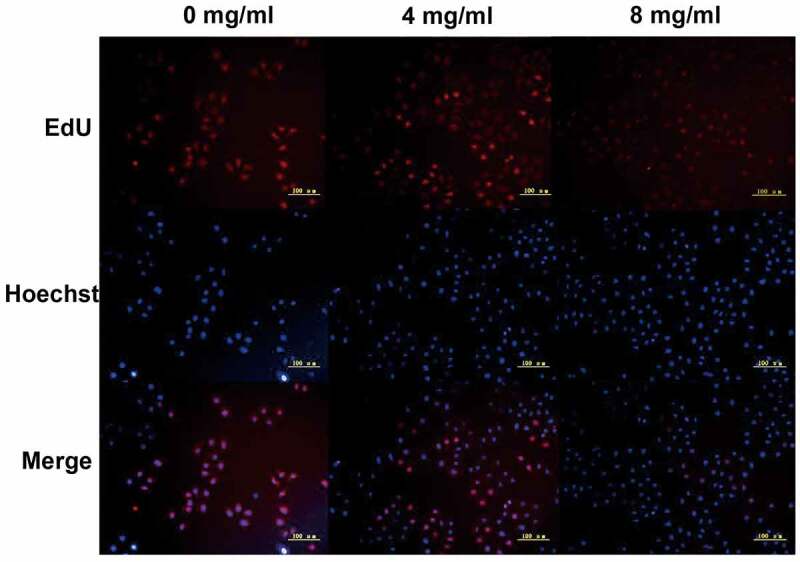
We used EdU staining to detect A549 cell line DNA replication. EdU positive cells was stained red and the nuclei were stained blue. The scale bars indicated 50 μm.

Subsequently, cell cycle arrest was detected. The proportion of S phase cells increased in a dose-dependent manner (S phase accumulation: A549 0 mg/ml: 3.87% ± 2.2%, 4 mg/ml: 10.9% ± 7.4%, 8 mg/ml: 18.1% ± 6.4%. H1299 0 mg/ml: 6.9% ± 3.2%, 4 mg/ml: 14.8% ± 4.6%, 8 mg/ml: 15.0% ± 6.5%. Apoptosis: A549 0 mg/ml: 1.05% ± 0.4%, 4 mg/ml: 12.44% ± 3.2%, 8 mg/ml: 35.14% ± 4.4%. H1299 0 mg/ml: 3.39% ± 1.2%, 4 mg/ml: 14.36% ± 3.2%, 8 mg/ml: 19.48% ± 2.4%. Figure 1(d)), suggesting that *Huaier* can arrest lung cancer cells in the S phase, which may be one of the mechanisms by which *Huaier* promotes apoptosis of lung cancer cells. The *Huaier*’s inhibitory effect on DNA replication was examined via the EdU assay in A549 cell line. With the increase of *Huaier* concentration, the number of EdU positive A549 cells decreased (Figure 2), and *Huaier* suppressed DNA replication.

To investigate effects of *Huaier* on EGFR mutant NSCLC cells, we employed PC9 cell line, an EGFR 19 exon deletion mutant lung adenocarcinoma cell line that are highly dependent on EGFR activation, for further study. In MTT assay, *Huaier* inhibited proliferation of PC9 cells in a concentration- manner (IC_50_: 24 h 7.98 mg/ml, Supplementary Material 2A). To further evaluate the inhibitory effect of *Huaier* on the growth of PC9 cells, we selected Gefitinib, a commonly used EGFR tyrosine kinase inhibitor (TKI) [28], as the standard reference drug. The results of the MTT assay showed that 8 mg/ml *Huaier* and 50 nM Gefitinib (a commonly used concentration of Gefitinib [29,30]) had similar inhibitory effects (Supplementary Material 2B), while the combination of *Huaier* and Gefitinib sharply decreased the viability of PC9 cells, and the extent of reduced cell viability by the combination therapy was significantly greater than either of the monotherapies. As shown in Supplementary Material 2C, similar results were observed in colony formation assay. PI-annexin V staining showed that apoptotic cells increased after the addition of Lyc and/or Gefitinib, with significant differences between the different treatments (Supplementary Material 2D).

In conclusion, these *in vitro* experiments showed that *Huaier* inhibited the growth of NSCLC cells.

### Identification of Huaier targets using bioinformatics

**Figure 3.**
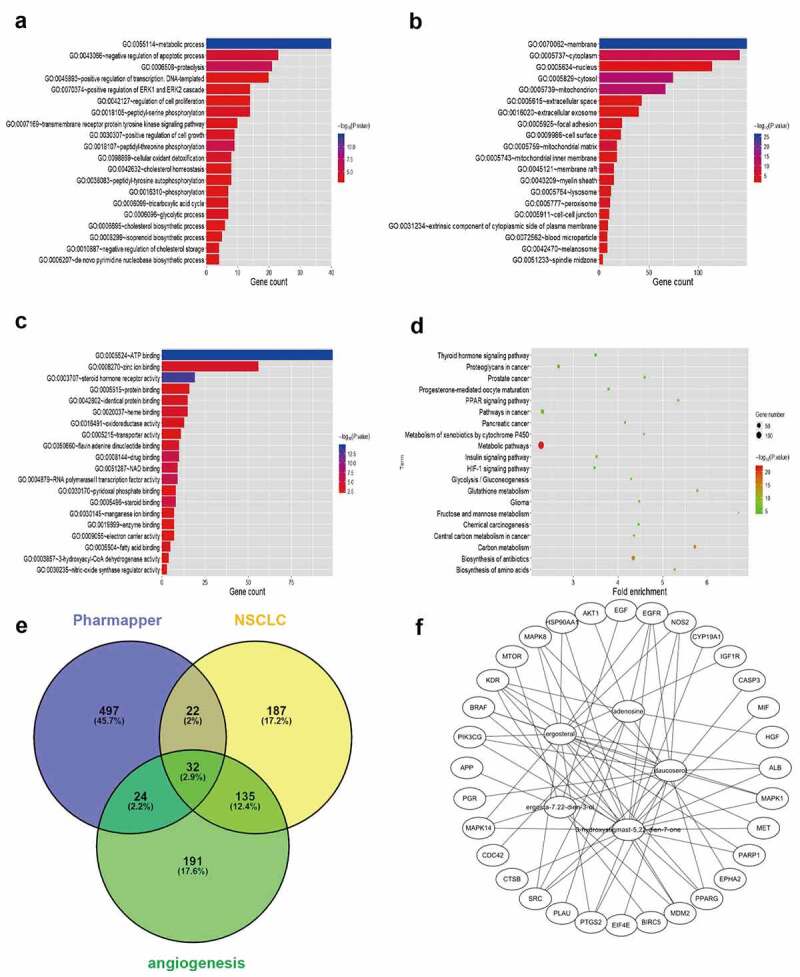
Bioinformatics prediction of *Huaier* targets. (a-c) Top 30 enriched Gene Ontology (GO) term enrichment analysis of *Huaier* targets. Gene counts were presented by the length of bars and the value of the minus log10 adjusted P value were presented by the gradation of color. (d) KEGG pathway enrichment analysis of *Huaier* targets with the top 20 enrichment scores. The gene counts were presented by the dot size and the value of minus log10 P value were presented by the gradation of color. (e) Identification of *Huaier*’s targets on NSCLC in Pharmmapper and CooLGen. A total of 32 common targets for drugs and diseases are obtained. (f) *Huaier* targets protein–protein interaction (PPI) network. Thirty-seven nodes (components of *Huaier* and targets) and 295 edges composed the PPI network.

Five structural formulas for *Huaier* components were uploaded to PharmMapper, where duplicate results were removed to obtain a total of 575 possible targets. The official symbol for the drug targets was obtained from UniProt. For a more in-depth understanding of the target protein, the GO function and KEGG pathway analyses were applied in the WebGestalt. The metabolic process was the most significant biological process (BP) term, membrane was the cellular component (CC) term with highest significance, and the most significant (MF) term was protein binding (Figure 3(a-c)). The KEGG pathway showed that the targets were involved in many metabolic pathways (Figure 3(d)). A total of 376 NSCLC-related targets were obtained from CooLGen. The *Huaier* targets were intersected with the NSCLC-related targets. Thirty-two possible targets for *Huaier* inhibition of lung cancer were obtained (Figure 3(e)). Among them, EGFR were the classical kinases driving lung cancer. Therefore, it was speculated that *Huaier* inhibits lung cancer through EGFR (Figure 3(f)).

### Huaier targets EGFR

**Figure 4.**
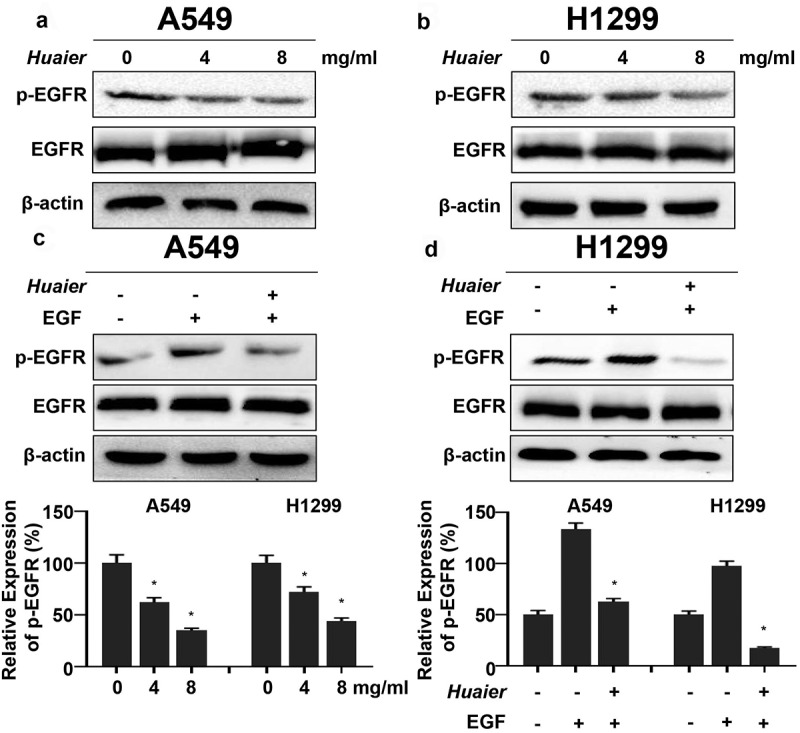
*Huaier* inhibits activation of EGFR. (a, b) Western blot was used to evaluate *Huaier*’s inhibitory effect on EGFR. β-actin was used as internal control. (c, d) A549 and H1299 cells were treated with *Huaier* and stimulated with EGF (25 ng/ml) for 20 min, then the western blot was applied to measure protein levels. Histograms of results (n = 3) are presented at the bottom. Data represents mean ± SD, *p < 0.05, n = 3. Uncropped western blot gels contained in Supplementary Material 4.

As mentioned above, EGFR was verified as *Huaier* targets verified using bioinformatics prediction. We applied western blotting to discover the protein expression changes to clarify the *Huaier*’s regulatory effect on the EGFR pathways. The total-EGFR expression did not change significantly after the *Huaier* treatment. The tyrosine 1068 phosphorylation of EGFR (p-EGFR) expression level was downregulated (Figure 4(a,b)). When lung cancer cells were treated with *Huaier* (6 mg/mL) and pretreated with EGF (25 ng/mL), it was evident that *Huaier* can inhibit the EGF-induced EGFR activation (Figure 4(c,d)). These results suggest that *Huaier* can inhibit the activation of EGFR.

### Huaier docks to EGFR

**Figure 5.**
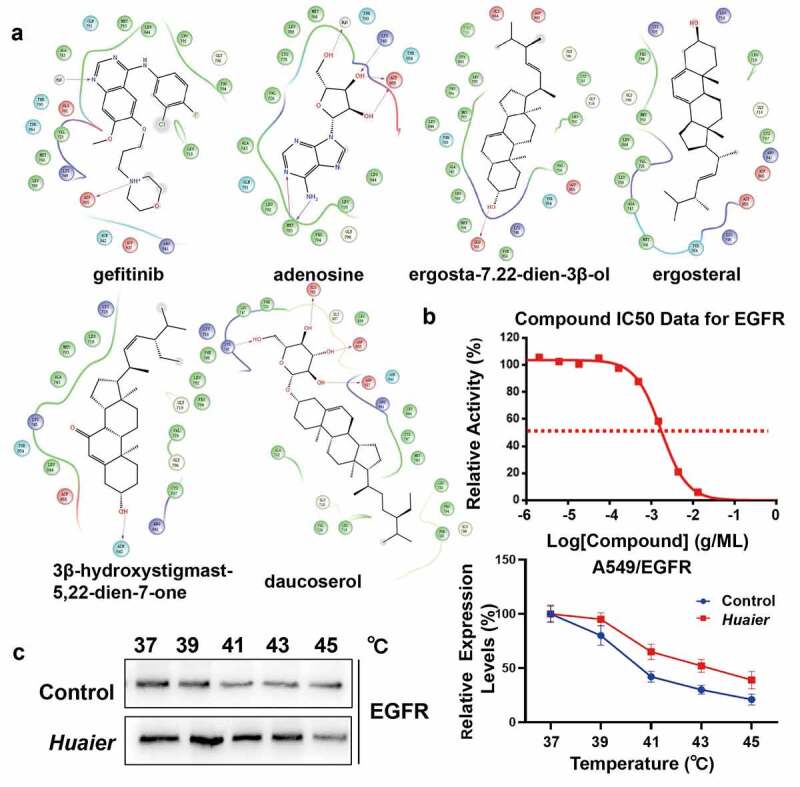
Virtual verification of *Huaier* targets by molecular docking. (a) Maestro 2D interactions between EGFR and gefitinib, adenosine, daucosterol, ergosterol, ergosta-7,22-dien-3β-ol and 3β-hydroxystigmast-5,22-dien-7-one. Residues in green spheres are hydrophobic, blue spheres are polar, red spheres are negatively charged, purple spheres are charged and light yellow spheres are glycine. The purple arrows and their directions represent hydrogen bonds between the ligand and the protein. The green line represents the π-π stacking arrangement seen between the aromatic core. (b) We conducted kinase activity assays to validate *Huaier*’s inhibition on EGFR kinase activity. Staurosporine, an effective PKC inhibitor, was chosen as positive control. (c) Cells were conducted by CETSAs, and the protein expression of EGFR was detected by western blot analysis. The line chart showed the relative expression level of EGFR.

Molecular docking is to judge the characteristics of receptors and the interaction between receptors and drug molecules. We used molecular docking to explore the specific mechanism of *Huaier* on the targets. To investigate geometrical complementarity and energy matching of *Huaier* and EGFR crystal structure, we chose gefitinib as a positive control. Previous reports on the five components of *Huaier* and molecular docking showed that adenosine and EGFR have the best binding capacity (Table 1), with a G-score of −8.805 kcal/mol, which is similar to that of gefitinib (G-score: −8.626 kcal/mol). Both adenosine and gefitinib can form hydrogen bonds with the EGFR Aspartate (Asp 555), suggesting that adenosine and gefitinib may have similar effects on EGFR (Figure 5(a)). Daucosterol and adenosine have similar binding with EGFR. Daucosterol also form hydrogen bonds with the EGFR Aspartate (Asp 555), with a G-score of −6.826 kcal/mol. Almost no steric hindrance exists between adenosine and EGFR. Similar to the molecular docking results, the kinase activity assay showed that *Huaier* inhibits EGFR with IC_50_ value of approximately 1.72 mg/mL (Figure 5(b)). Subsequently, we performed CETSAs to determine the docking EGFR protein and *Huaier in cellulo*. As shown in Figure 5(c), the EGFR melting curve was shifted after *Huaier* administration. In order to explore the effective components of *Huaier* inhibit targets preliminarily, we conducted kinase activity assays between adenosine and EGFR, according to the molecular docking results. The results showed that adenosine didn’t inhibit EGFR activity. Another component in *Huaier*, i.e., daucosterol, also docked to EGFR. Kinase activity assay showed that daucosterol inhibited EGFR with an IC_50_ of approximately 3.12*10^-7 M (Supplementary Material 5A). Overall, molecular docking and kinase activity assay showed that *Huaier* binds well with EGFR.

**Table 1.** Binding score of gefitinib (positive control)/huaier and EGFR (kcal/mol)

gefitinib	adenosine	ergosta-7.22-dien-3β-ol	ergosteral	3β-hydroxystigmast-5,22-dien-7-one	daucoserol
-8.233	−8.393	−5.648	−4.206	−5.142	−4.826

### Huaier inhibits NSCLC in vivo

**Figure 6.**
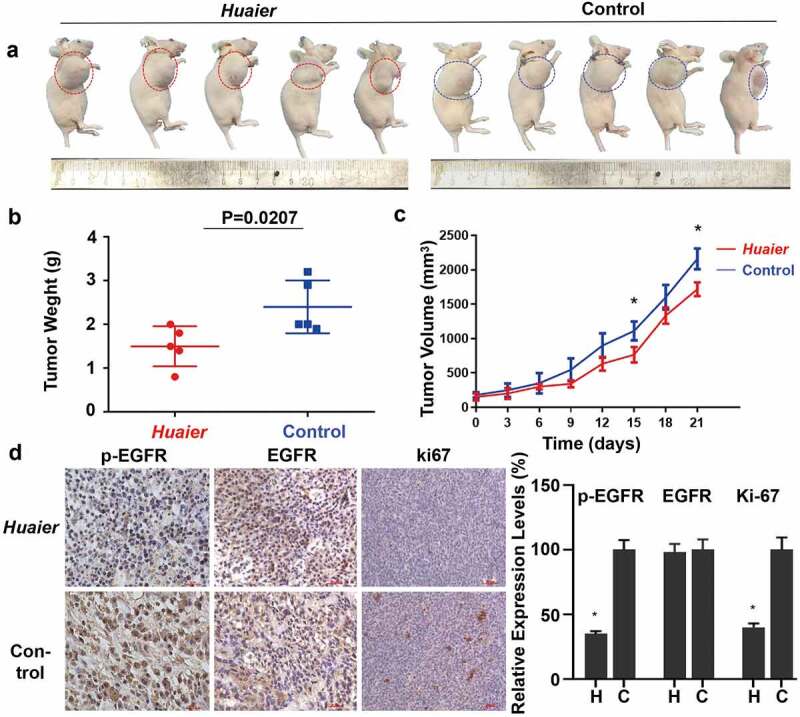
*Huaier* inhibits NSCLC *in vivo*. (a) Representative images of the tumor treated with *Huaier* or not. (b) In the end of the experiment, we sacrificed mice and obtained tumor weight. Tumor weight (in g) on the day 21 was shown in the graph. (c) We recorded and compared tumor volumes every three days. Tumor volume (in cm^3^) over a total of 21 days was shown in the graph. Data represents mean ± SD, *p < 0.05. (d) We used immunohistochemistry to analyze p/t-EGFR and Ki-67 expression. Data represents mean ± SD, *p < 0.05. The high quality/resolution H&E and immunohistochemistry images contained in Supplementary Material 6.

To validate the results of cell experiments, a xenotransplantation model of human NSCLC in mice was established in mice using A549 cells. *Huaier* was administered for 3 weeks before the mice were sacrificed. The volume of the xenograft tumors decreased after the *Huaier* administration compared with the control group (Figure 6(a)). The xenograft tumors was also significantly lighter than that of the control group. The xenograft tumors average weight was 1.5 ± 0.41 g in the *Huaier* treatment group and was 2.4 ± 0.54 g in the placebo treatment group (Figure 6(b)). The transplanted tumors growth rate in the *Huaier* treatment group was slower than that in the placebo treatment group. After approximately 18 days, transplanted tumors size between two groups differed significantly (Figure 6(c)). The expression levels of p/t-EGFR and cell proliferation marker Ki-67 were detected using immunohistochemistry to explore *Huaier*’s mechanism on tumor growth inhibition. *Huaier* reduced the expression of p-EGFR and proliferation, while it had no effect on total-EGFR expression (Figure 6(d)). During the experiment, the weight changes in mice were regularly monitored. The average weights of the two groups were similar (Supplementary Material 5B). The mice treated with *Huaier* had no clinical symptoms of disease and discomfort, and no obvious drug-induced liver and kidney damage (Supplementary Material 5C) was found by H&E staining, which indicated good *Huaier* tolerance. These results demonstrate that *Huaier* is effective and safe *in vivo*.

## Discussion

One of the leading cause of cancer-related death worldwide is lung cancer and its effective treatment is still scarce. Approximately 80% of traditional Chinese medicines come from plants, among which fungal Chinese medicines play an important role. The *Huaier* fungus extract was discovered during a treatment of hepatocellular ascites. But, how *Huaier* works on lung cancer remains unclear. Chen [31] found that *Huaier* inhibited lung cancer proliferation and metastasis through MTDH, JAK2/STAT3, and MAPK. In addition, other studies [32] have shown that *Huaier* regulates lung cancer sensitivity to cisplatin via the mTOR pathway. In addition, several microRNAs have also been identified as targets of *Huaier* (e.g. let-7d-5p [33], miRNAs-203 [34] and miRNAs-26b-5p [35]), suggesting that *Huaier* has multitarget and multipathway pharmacological effects in anti-cancer thearapy. The specific mechanism involved in *Huaier*’s inhibition of lung cancer is still not clear. In this study, we found for the first time that *Huaier* inhibited EGFR’s activiation by binding to the ATP-pocket directly.

Investigation of pharmacodynamic substances and mechanisms by bioinformatics can reveal new research directions and ideas for traditional experimental research methods [36]. Molecular docking is a process of recognition by space and energy matching between proteins and drugs, which can also simulate drug-target binding [37]. In this study, nearly 600 *Huaier* targets were obtained through the PharmMapper database. The GO and KEGG pathway enrichment analyses showed these targets were involved in many tumor-associated biological processes. Using screening, EGFR was predicted to be *Huaier*’s targets for NSCLC inhibition. Through virtual verification of molecular docking, it was shown that EGFR can bind to *Huaier*. Additionally, using molecular docking, we found that *Huaier* active ingredients not only bind to wild-type EGFR, but also bind to exon 21 mutant EGFR (L858R) (data not shown). Therefore, we conclude that *Huaier* can also inhibit the activity of mutant EGFR, but this observation needs to be confirmed by further experiments. We speculated that daucosterol may be the effective components of *Huaier* in inhibition of EGFR. Adenosine and EGFR had a higher G-score, but failed to inhibit EGFR activity, while daucosterol had a lower G-score but inhibited EGFR activity. This result may be attributed to the higher molecular weight (MW) of daucosterol (adenosine: 267.24, daucosterol: 576.85), which may be able to better enter the ATP pocket of EGFR. On the other hand, it is limited to evaluate the binding affinity of ligand and receptor by Glide score or docking score.

The imbalance of proliferation and apoptosis is one of the characteristics of cancer, which indicates that inhibiting proliferation and promoting apoptosis is an important anti-cancer therapy strategy. In this study, it was found *Huaier* can inhibit the growth of A549 and H1299 cell lines. EGFR is a tumor proliferation-related molecule. *Huaier* can inhibit the activation of EGFR. The molecular docking results showed that the binding sites of *Huaier*’s active components for EGFR were located near the tyrosine 1068 phosphorylation sites, which may be one of the mechanisms of *Huaier*’s inhibition of Growth Factor Receptor-Bound protein 2 (GRB2), an adaptor protein that forms stable complexes with tyrosine-phosphorylated EGFR, activating intracellular signaling pathways [38]. In the next study, we will explore if *Huaier* influence expression or location of GRB2.

## Conclusion

TCM has unique advantages in cancer treatment, but its effective substances and mechanisms are not yet clear. In this study, using bioinformatics prediction, molecular docking, and *in vitro* and *in vivo* experiments, it was revealed that *Huaier* attenuated the EGFR activation. We preliminarily showed that daucosterol is the component that inhibit EGFR, but this needs further demonstration. Possible *Huaier* impact on other targets, especially other tyrosine kinase, needs to be further investigated. We look forward to a large sample of clinical trials to verify the efficacy of *Huaier* on NSCLC patients for improved lung cancer patients’ survival.

## Supplementary Material

Supplemental Material

## Data Availability

All the data generated and/or analyzed during this study are included in this published article (and its supplementary information files), and other datasets will be available from the corresponding author on reasonable request.
