# Supplementary material for: An extraction from Trametes robiniophila Murr. (Huaier) inhibits non-small cell lung cancer proliferation via targeting to epidermal growth factor receptor
Source: Bioengineered. 2022 Apr 26;13(4):10931–43. doi: 10.1080/21655979.2022.2066757 (PMC9162005; doi:10.1080/21655979.2022.2066757)
Supplement: Supplemental Material [file KBIE_A_2066757_SM7260.zip › supplementary/downloadFromZipFile.pdf]

中国医科大学实验动物福利与伦理审查表(供课题申报)

China Medical University Application for Laboratory Animal Welfare and Ethical review (Issue declaration)

申请日期：2020.02.05　　**批准日期：**　　**批准文号：**
Appl.Date:　　Appr.Date:　　IACUC Issue No.

<table>
<tr><td colspan="8">一、项目与人员信息</td></tr>
<tr><td>课题名称
Program</td><td colspan="7">槐耳提取物抑制非小细胞肺癌增殖、迁移侵袭和血管新生的机制研究
Mechanism of inhibition of growth, migration, invasion and angiogenesis in non-small cell lung cancer by Huaier extract</td></tr>
<tr><td>课题负责人
Name of rincipal Investigator</td><td>吕飞</td><td>课题来源
Sponsor</td><td colspan="5">自选</td></tr>
<tr><td>单位/科室
Department</td><td>盛京医院/肿瘤三科</td><td>电　话
Tel. No</td><td>18940253307</td><td>信箱
E-mail</td><td colspan="3">627734249@qq.com</td></tr>
<tr><td>课题参与者
Project participants</td><td colspan="7">Lvfei</td></tr>
<tr><td colspan="8">参与动物实验操作人员姓名、培训编号　Name and certificate number</td></tr>
<tr><td>姓　名
Name</td><td>单位/科室
Department</td><td>培训编号
License No.</td><td>电话
Tel. No</td><td colspan="4">信箱
E-mail</td></tr>
<tr><td>吕飞</td><td>盛京医院/肿瘤三科</td><td>201907099</td><td>18940253307</td><td colspan="4">627734249@qq.com</td></tr>
<tr><td colspan="8">二、实验动物信息</td></tr>
<tr><td>动物来源
Animal origin</td><td colspan="3">☑ 实验动物部
Department of Laboratory Animals
□ 采购/赠予(Procurement / Gift)
（具体单位名称：______________ ）
□ 国外引进(International purchase)</td><td colspan="2">质量合格证
Certification of fitness</td><td colspan="2">☑ 有
Yes
□ 无
No</td></tr>
<tr><td>品种/品系
breed/strain</td><td colspan="3">□ 大鼠(Rat)________________
☑ 小鼠(Mouse) ___裸鼠_______
□ 豚鼠(Guinea pig)_____□ 兔(rabbit) _____
□ 犬 (Dog)_________□ 猪(Pig) _________
□ 猴（Monkey）____□其它（Other） ____</td><td colspan="2">动物级别
Grade</td><td colspan="2">□普通（CV）
□清洁（CL）
☑SPF
□无菌动物（GF）</td></tr>
<tr><td>数量（只）
Number (♀; ♂)</td><td>雌（♀）20 ;
雄（♂）________;</td><td>周/月龄
W/M Age</td><td>4-6w</td><td colspan="2">体重（g）
Weight</td><td colspan="2">14-20g</td></tr>
<tr><td colspan="8">拟实验时间：2021年 7月1日 至 2021 年12月 31 日
Experimental period</td></tr>
<tr><td colspan="8">三、研究项目信息</td></tr>
</table>

1. 动物实验项目的目的、必要性、意义和如何设计以达成研究目标<br>Experimental objective, necessity and significance and how the program has been designed to achieve the objectives of the research<br>槐耳提取物作为中药的一种，已经广泛用以三阴性乳腺癌和肝细胞癌的医治中。槐耳提取物对非小细胞肺癌的作用及其机制却尚不明晰，本研究拟明确槐耳抑制非小细胞肺癌的作用机制，其目的是为非小细胞肺癌（NSCLC）的辅助医治提供更多的可供选择药物。
2. 说明实验对动物可能造成的所有可预期的伤害，包括每个实验方案中可能产生副作用的细节以及采取的防控措施<br>Description of the overall harms expected to be experienced by the animals – including details of the likely adverse effects of each protocol and the steps which will be taken to control these adverse effects<br>经尾静脉注射肿瘤细胞，可能会损伤小鼠<br>措施：学习并熟练掌握注射方法
3. 动物替代、减少动物用量、降低动物痛苦伤害的主要措施<br>Major measure for 3Rs<br>尽量减少动物的使用量，尽量改善动物实验环境，尽量减少动物痛苦，必要时应使用麻醉剂，处死动物应实施安乐死
4. 仁慈终点或实验终结的指标<br>Humane endpoint or experimental terminative indicator<br>转移瘤的大小及数量；小鼠存活时间
5. 动物死亡处理<br>Death conduct<br>□ CO_2窒息 CO_2 suffocated　　□ 麻醉后放血致死 Exsanguinations with anesthesia<br>☑ 颈椎脱臼致死 Cervical dislocation　　□ 麻醉过量致死 Anesthesia overdose<br>□ 其他 Others, detailed description
6. 非处死动物的处置方式<br>Not for the death of the animal disposition<br>☑ 继续使用 Continue to use　　□ 保存的机构 Save in the agency<br>□ 放生野外 Release to the wild　　□ 其他，详细说明 Others, detailed description
7. 是否使用有毒（害）物质 （感染、放射、化学毒、其他）<br>Poisonous (harmful) material(infection, radiate, chemical poison and other)being used

□ 是　　　　☑ 否
Yes　　　　no
说明：
Declare

声明：

1. 我将自觉遵守实验动物福利伦理相关法规和各项规定，同意接受伦理委员会和实验动物室管理者的监督与检查；
2. 本人保证本申请表中所填内容真实、详尽和易懂。

Declaration：

1. I will abide by the law and regulation stipulation, and accept the supervision and inspection by the committee and laboratory animal department.
2. The information I have given is accurate, detailed and comprehensive.

声明人：课题负责人签（章）

Declarant：Signature (stamp) of PI

动物实验负责人签（章）

Signature (stamp) of Director of animal experiment

2020 年2月 5 日

福利伦理委员会审批意见

Approval opinion

☑ 批准　　　　□ 不批准

Approval　　　　Not approve

IACUC
陈克研
合格

指定负责人签（章）：

Authorized Personnel Signature（Stamp）

2020 年 3月 1 日

申报说明：
Notice

1. 一个项目填写一份申请表。
   A project should fill out one IACUC application.
2. 本表内容需填写完整，发送至邮箱 iacuc@cmu.edu.cn，审批，周期为 10 个工作日。
   Application form should be fill in completely，then it need to be sent to E-mail：iacuc@cmu.edu.cn. We guarantee to reply in 10 working days.
3. 批准后，课题负责人签字，纸质版交至 IACUC 办公室。
   After being approved，the PI signed hard copy need to be submitted to the office of IACUC.
