# Supplementary material for: An extraction from Trametes robiniophila Murr. (Huaier) inhibits non-small cell lung cancer proliferation via targeting to epidermal growth factor receptor
Source: Bioengineered. 2022 Apr 26;13(4):10931–43. doi: 10.1080/21655979.2022.2066757 (PMC9162005; doi:10.1080/21655979.2022.2066757)
Supplement: Supplemental Material [file KBIE_A_2066757_SM7260.zip › supplementary/Supplementary Material 4.docx]

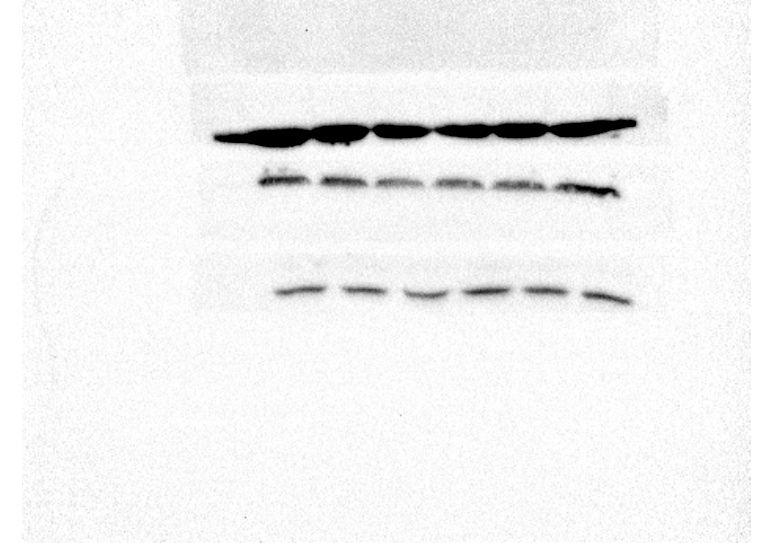


**Figure 3A, p-EGFR**


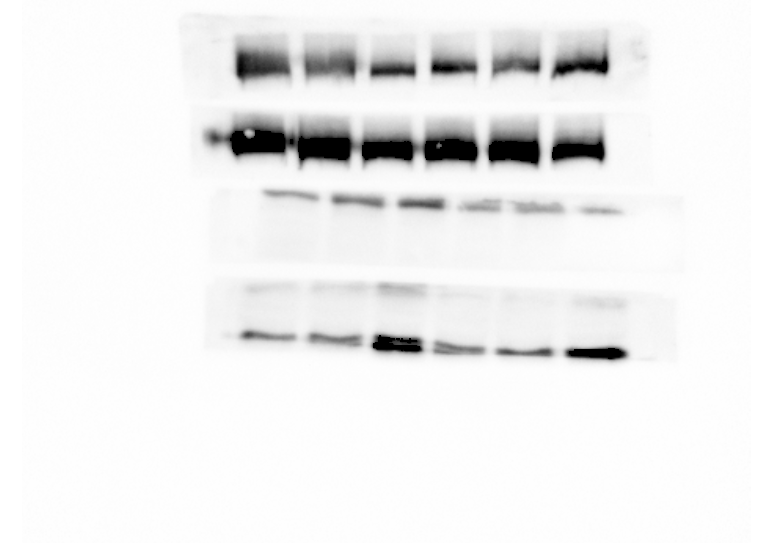


**Figure 3A, EGFR**


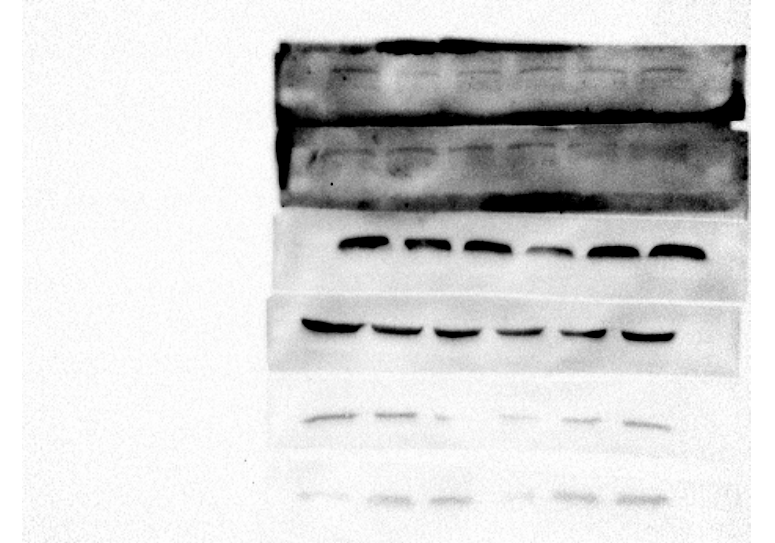


**Figure 3A, β-actin**


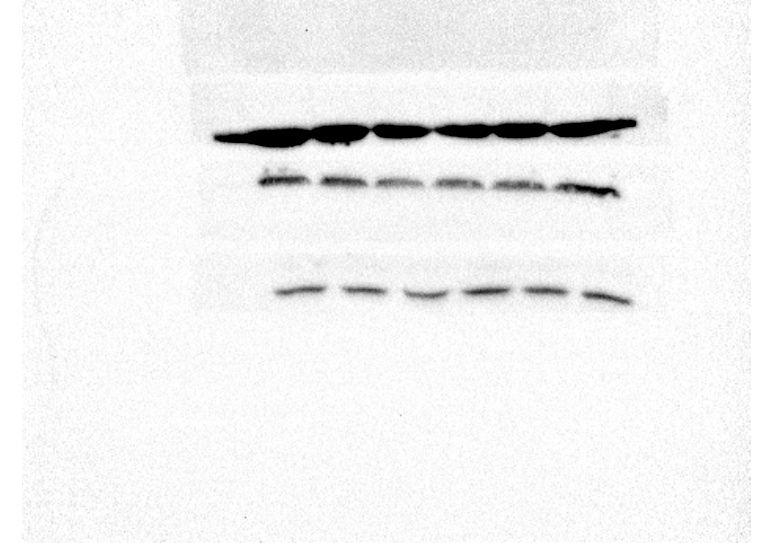


**Figure 3B, p-EGFR**


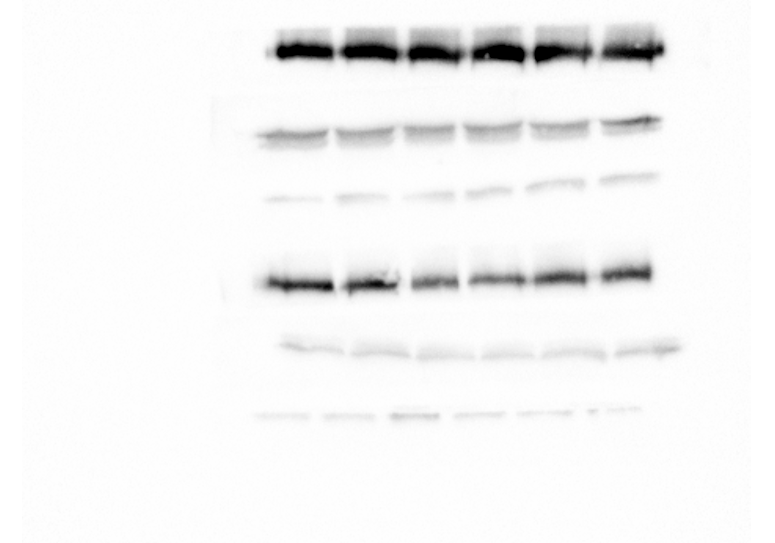


**Figure 3B, EGFR**


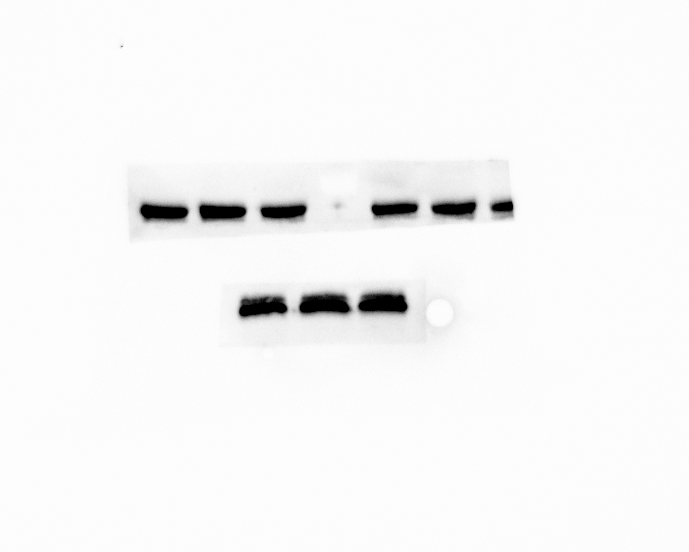


**Figure 3B, β-actin**


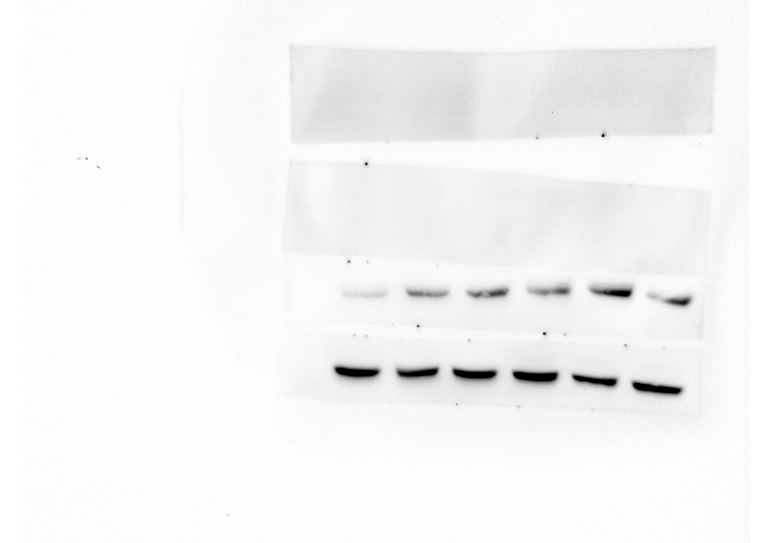


**Figure 3C, p-EGFR**


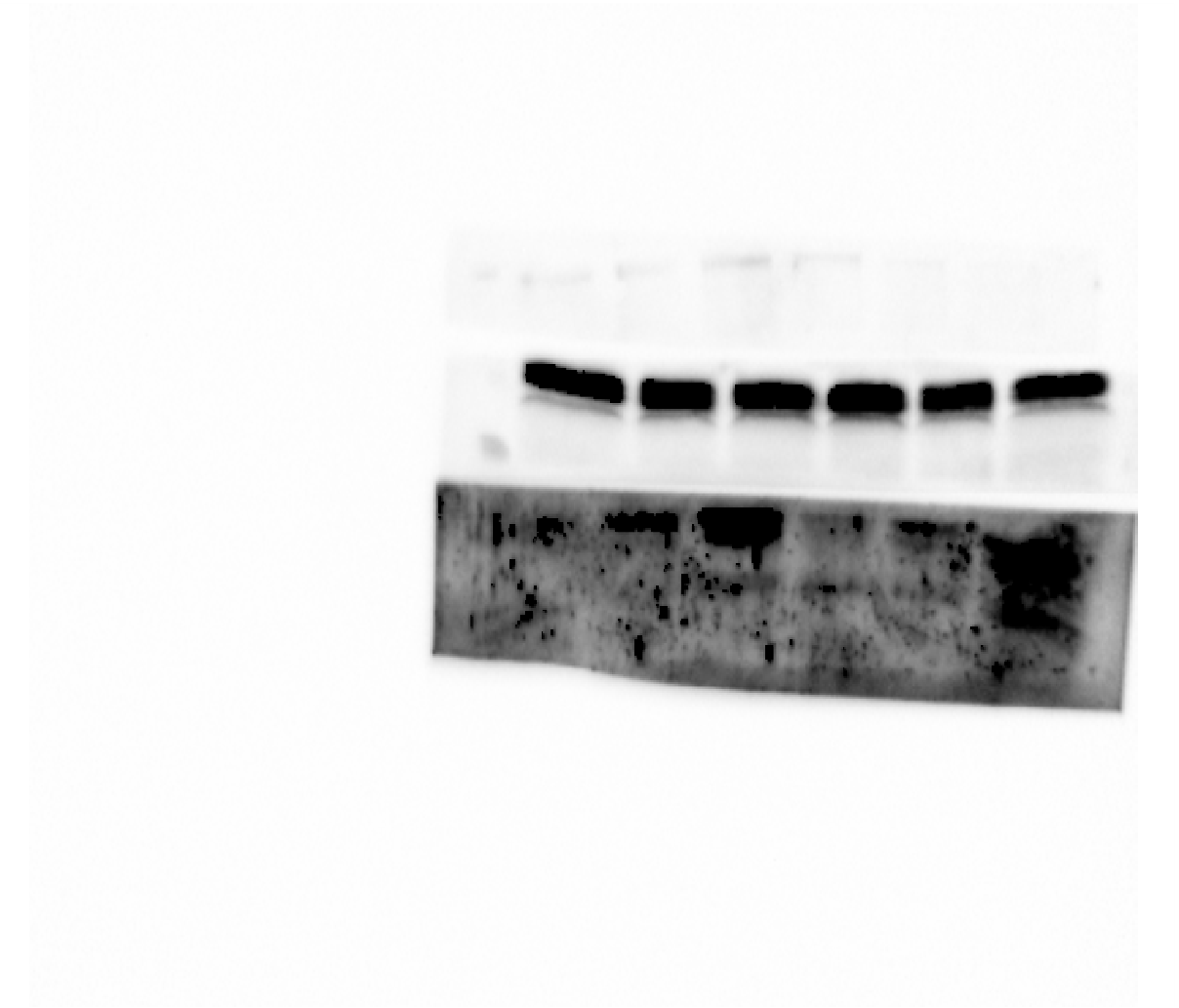


**Figure 3C, EGFR**


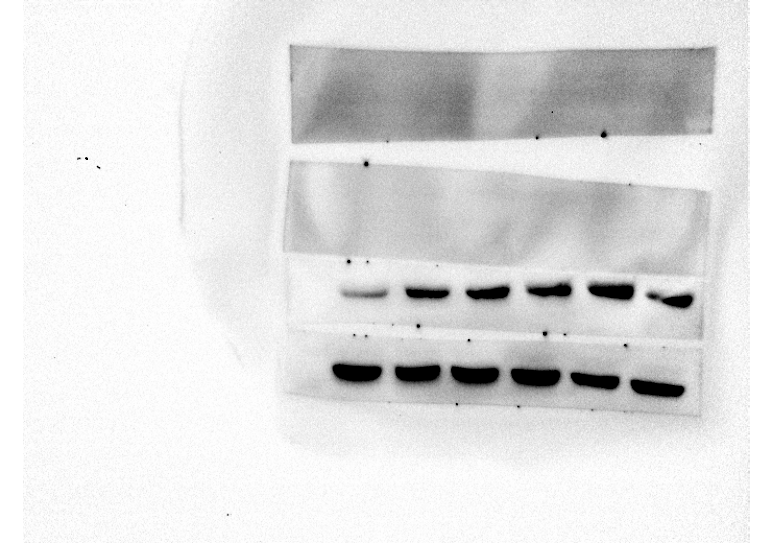


**Figure 3C, β-actin**


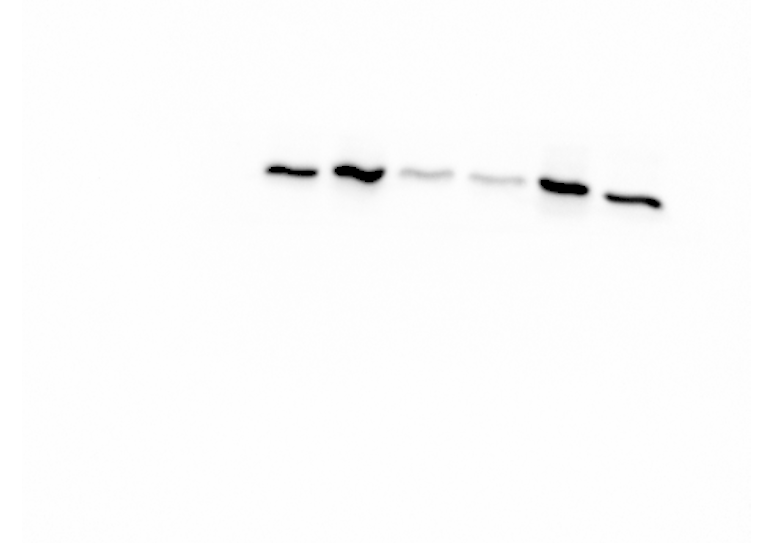


**Figure 3D, p-EGFR**


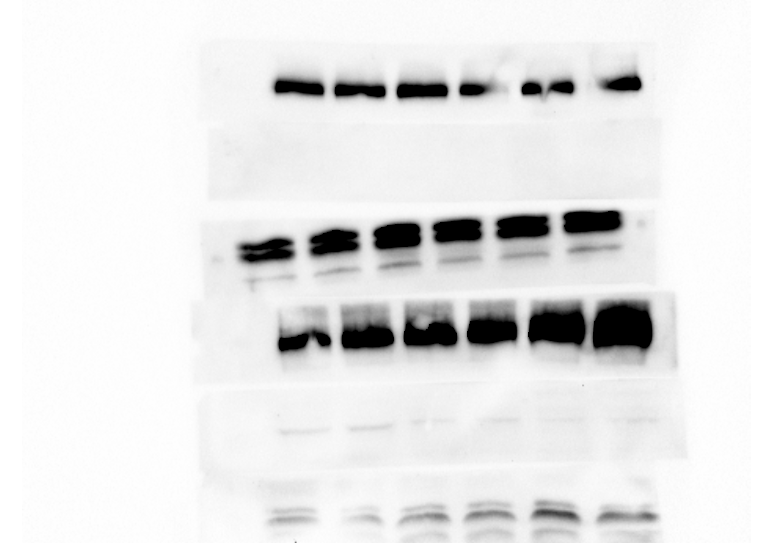


**Figure 3D, EGFR**


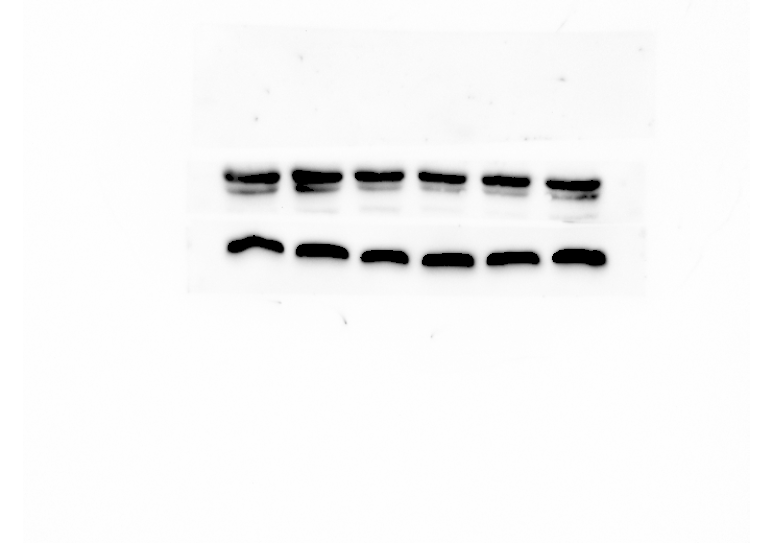


**Figure 3D, β-actin**
