# Supplementary figures and images for: An extraction from Trametes robiniophila Murr. (Huaier) inhibits non-small cell lung cancer proliferation via targeting to epidermal growth factor receptor
Source: Bioengineered. 2022 Apr 26;13(4):10931–43. doi: 10.1080/21655979.2022.2066757 (PMC9162005; doi:10.1080/21655979.2022.2066757)

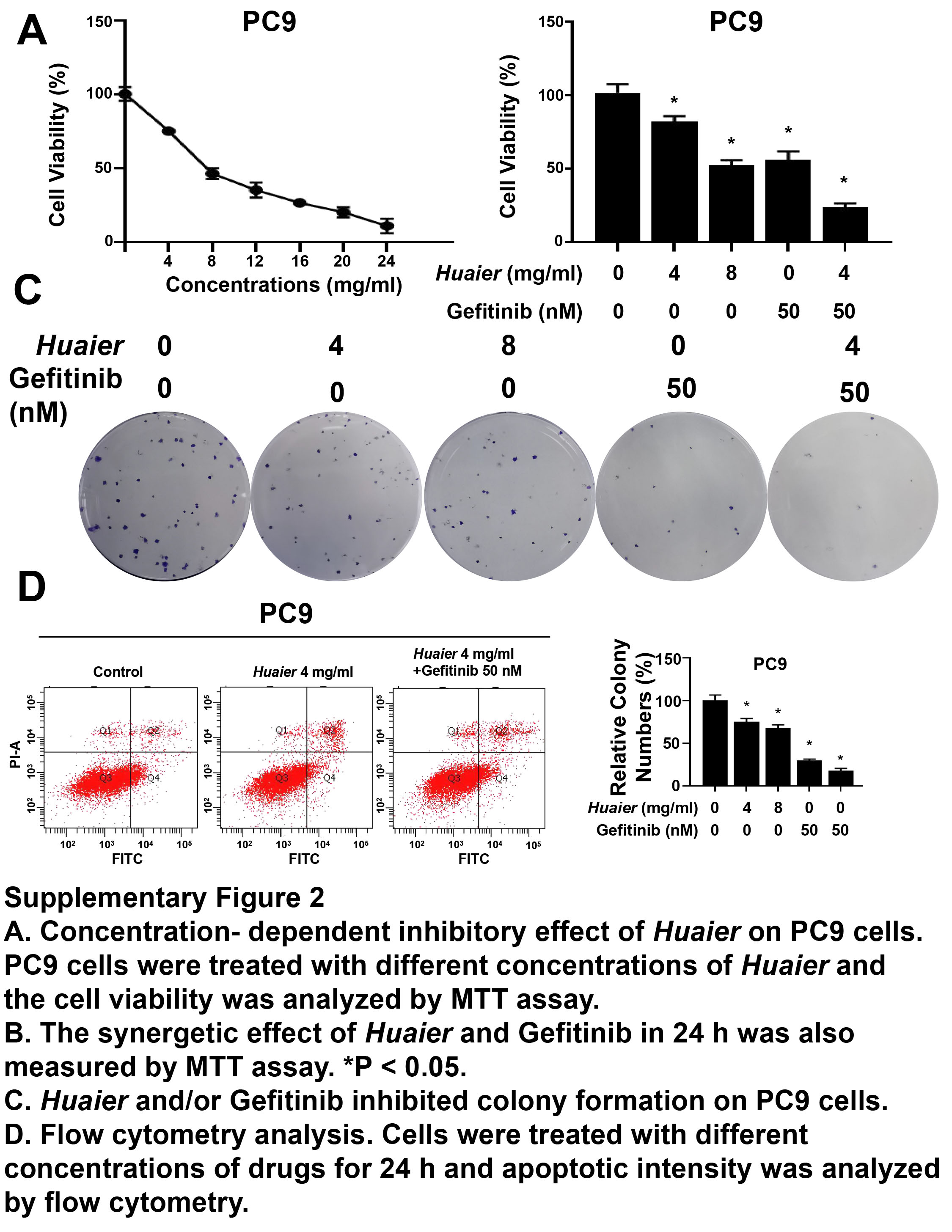

Supplement: Supplemental Material [file KBIE_A_2066757_SM7260.zip › supplementary/Supplementary Material 2.jpg]

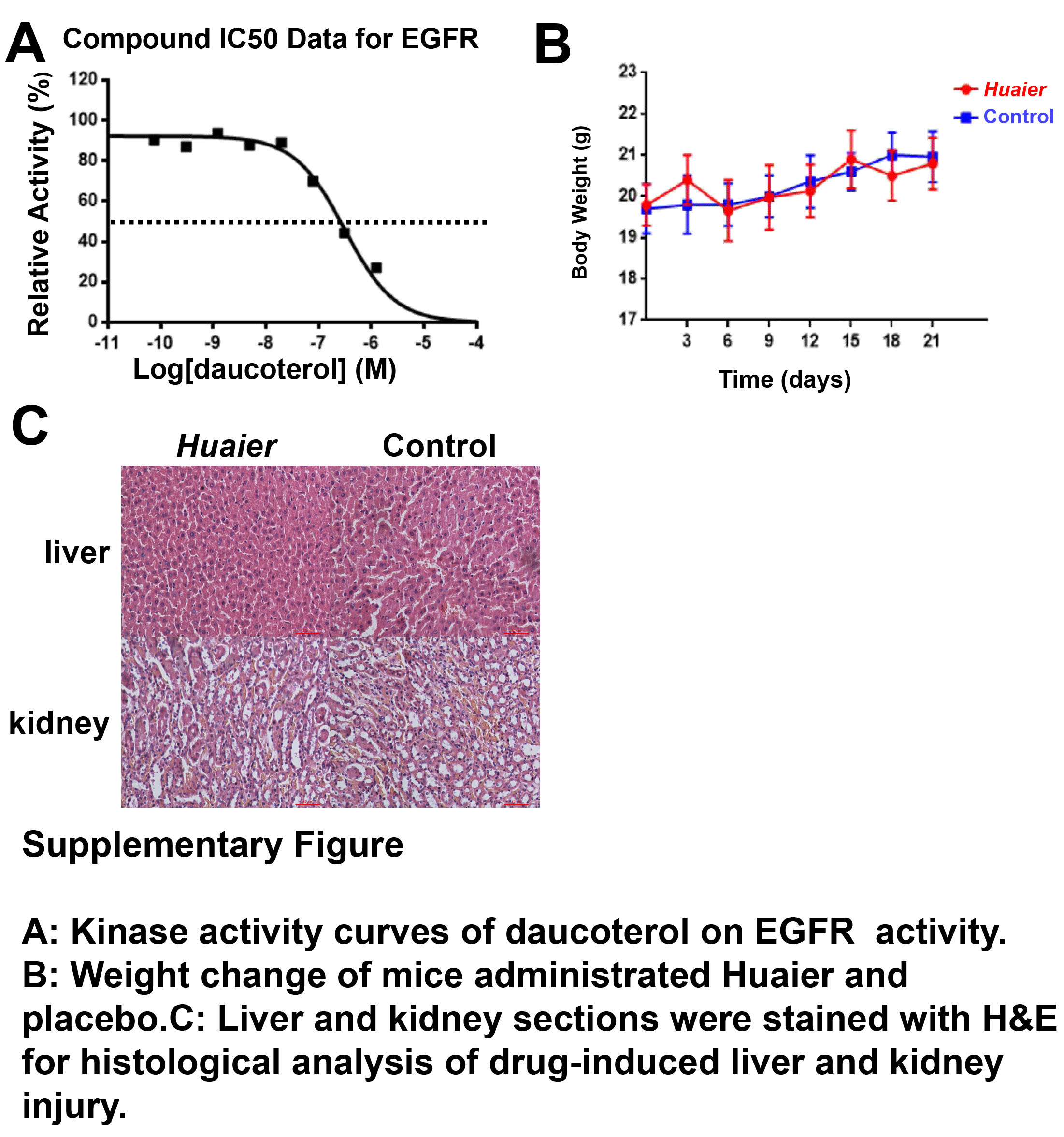

Supplement: Supplemental Material [file KBIE_A_2066757_SM7260.zip › supplementary/Supplementary Material 5.jpg]
